# Application of Improved UNet and EnglightenGAN for Segmentation and Reconstruction of In Situ Roots

**DOI:** 10.34133/plantphenomics.0066

**Published:** 2023-07-06

**Authors:** Qiushi Yu, Jingqi Wang, Hui Tang, Jiaxi Zhang, Wenjie Zhang, Liantao Liu, Nan Wang

**Affiliations:** ^1^ College of Mechanical and Electrical Engineering, Hebei Agricultural University, 071000, Baoding, China.; ^2^ College of Agronomy, Hebei Agricultural University, 071000, Baoding, China.

## Abstract

The root is an important organ for crops to absorb water and nutrients. Complete and accurate acquisition of root phenotype information is important in root phenomics research. The in situ root research method can obtain root images without destroying the roots. In the image, some of the roots are vulnerable to soil shading, which severely fractures the root system and diminishes its structural integrity. The methods of ensuring the integrity of in situ root identification and establishing in situ root image phenotypic restoration remain to be explored. Therefore, based on the in situ root image of cotton, this study proposes a root segmentation and reconstruction strategy, improves the UNet model, and achieves precise segmentation. It also adjusts the weight parameters of EnlightenGAN to achieve complete reconstruction and employs transfer learning to implement enhanced segmentation using the results of the former two. The research results show that the improved UNet model has an accuracy of 99.2%, mIOU of 87.03%, and F1 of 92.63%. The root reconstructed by EnlightenGAN after direct segmentation has an effective reconstruction ratio of 92.46%. This study enables a transition from supervised to unsupervised training of root system reconstruction by designing a combination strategy of segmentation and reconstruction network. It achieves the integrity restoration of in situ root system pictures and offers a fresh approach to studying the phenotypic of in situ root systems, also realizes the restoration of the integrity of the in situ root image, and provides a new method for in situ root phenotype study.

## Introduction

The root is an important organ for plants to absorb water and nutrients and plays a vital role in plant growth and productivity. Excellent root development is also the basis for high-quality and high-yield crops [1–3]. Studying root phenotypes is an important basis for exploring the growth and development of crops and breeding excellent crop varieties.

Traditional root acquisition techniques, like root drilling and sectioning, waste time, labor and material resources, and the washing process can cause issues like damage to the root architecture and the loss of fine root segments. In situ root research methods (microroot window method and root box method) are being accepted by researchers with the innovation of image processing technology [4,5]. The in situ root imaging method originated from the microroot canal method [6], which refers to the acquisition of root images by inserting a glass tube into the soil and photographing the root in contact with the glass tube wall. This method has a large number of technologies deployed in the field [7], but it has a low collection efficiency and a low root image resolution.

Three-dimensional root architecture studies are also recognized in in situ root studies. X-ray tomography and Magnetic resonance imaging have been applied to study root phenotypes in situ [8]. X-ray tomography technology uses the different attenuation characteristics of x-rays after passing through the soil and roots to scan root images and finally obtains a more complete root space image [9–11]. Magnetic resonance imaging technology is to obtain the nuclear magnetic resonance information of different positions of the object in the magnetic field by emitting electromagnetic waves and using the computer to reconstruct the internal image of the object [12] to obtain the image of the root [13–15]. However, the imaging time of these 2 techniques is long, the resolution is low, and the root with a diameter of less than 400 μm cannot be identified [16]. Also, the cost of the equipment is high, and it is easily disturbed by environmental factors.

The digital equipment imaging method is to use a smartphone, a digital camera, or a scanner in combination with a cultivation device to jointly complete the root image acquisition method. It is possible to dynamically collect high-resolution in situ root images without changing the soil environment and affecting root growth status, which is conducive to improving the efficiency of root architecture segmentation and quantitative analysis [17]. Currently, this method has been extensively reported [18–20]. Based on obtaining high-resolution in situ root images, it is difficult to accurately and efficiently distinguish roots from the soil and other impurities [21].

Traditional in situ root image recognition methods include manual drawing, semiautomatic interactive recognition, and fully automatic threshold segmentation. Among them, the artificial drawing method is highly dependent on human subjectivity, with large errors and low efficiency [22,23]. Although the semiautomatic interactive identification method has improved efficiency, it still relies too much on the operator's subjective ability to distinguish roots and his own experience. The automatic threshold segmentation method has obvious improvements in efficiency and objectivity, but there are problems with large soil noise interference and large recognition errors.

Compared with traditional methods, in situ root image recognition based on deep learning is more conducive to mining the deep features of the target. SegRoot [24] based on SegNet [25] can distinguish dark soil from roots, but underfitting may occur in some cases. Soybean nodule acquisition pipeline [26] based on UNet [27] and RetinaNet [28] can accurately detect nodules on soybean roots and segment the main root. Gaggion et al. [29] proposed ChronoRoot integrating UNet, ResUNet, DSResUNet, SegNet, and DeeplabV3+ in 2021. The model principle is to average the segmentation results of the integrated model, which can effectively realize the architecture and integration of multiple models.

The root image recognition based on deep learning also found shortcomings, such as the root segmentation process of the semantic segmentation network, which is prone to root breaking and root fragmentation. Or part of the root is covered by the soil in the in situ root image, and the fragmentation of the root also occurs. This is not conducive to the collection of root phenotypic characteristics for example the root architecture. Eliminating the fragmentation of the root and improving the integrity of the root are essential supporting means for in situ root research. The improvement of image restoration methods provides the conditions for a more accurate acquisition of in situ root images. At present, the method of repairing pictures based on generative adversarial network (GAN) has been widely used, such as Patch-GAN and DE-GAN for face repair [30,31]; cGAN for data augmentation in mustard [32]; in terms of root repair, SRGAN can repair root loss caused by blurred and low-resolution root images [33]. However, this method also has limitations. For example, Arabidopsis roots growing in transparent media can be restored, but it does not suit for the plant roots growing in soil. The roots in this transparent medium can judge the location of the roots even if the resolution is not high. But for the in situ root in the soil matrix, the occlusion of the soil makes root restoration a problem from scratch.

EnlightenGAN belongs to an efficient and unsupervised generative adversarial network. By paying attention to the details of the dark part, we can use the normalized grayscale image as the attention map. Through the generator of the UNet structure, the image after the illumination enhancement is obtained and then trained by the multiscale discriminator, with the process unsupervised [34]. In this study, EnlightenGAN is proposed to be used for root reconstruction. By varying the light intensity of the target area, a principle similar to that of the root zone is generated.

The RhizoPot platform previously developed by our laboratory has been reported [35], which can realize a nondestructive collection of complete root images through scanners and RhizoPot growth devices. Based on the improvement of DeeplabV3+, our research team proved the possibility of accurately segmenting the root in the early stage, but there is a problem the root diameter and surface area are far from the actual value in the root phenotype analysis results [36]. In the follow-up, the research team conducted continuous research on the accurate segmentation method of roots [37,38], which improved the accuracy of in situ root segmentation in the soil background, but the small pieces covered by the soil cannot be identified. The purpose of this article is to repair the root covered by the soil and solve the phenomenon that the deep learning segmentation of the in situ root cannot be fully fitted. To this end, we improved the UNet model, integrated the convolutional block attention module (CBAM) [39] attention mechanism and subpixel convolution into the semantic segmentation network; modified the parameters of EnlightenGAN so that it can accurately generate images of reconstructed roots; and demonstrated the semantic segmentation network and generation adversarial network. The combination scheme of the network clarifies the role of different schemes and proves the possibility of unsupervised learning of root reconstruction. At the same time, it analyzes the phenotype data of the network and makes a prospect for further improving the network to adapt to complex roots.

## Materials and Methods

### The original root dataset

In the earlier papers of this experimental group [35,40,41], the operation of the image acquisition apparatus and the procedure for cotton seed germination were discussed. With an Epson scanner V39 (Epson lnc., Suwa-shi, Nagano, Japan) and an image resolution of 10,200 × 14,039 pixels, we planted germinated cotton plants in 8 groups of RhizoPot devices and acquired images of the roots of cotton seedlings for 110 consecutive days. The image resolution was 10,200 × 14,039 pixels, using a 1,200-dpi setting. The dataset for the annotation process consisted of 125 photos with complete root growth, distinct structure, and no noise.

Using the lasso tool in Adobe Photoshop CC (Adobe Inc., San Jose, CA, United States), seasoned agronomists annotated images. The remaining pixels were eventually labeled black after all of the roots were stored in a new layer with a white label. A single image takes roughly 4.5 h to annotate.

For network training, a total of 100 sets of training and validation data are chosen at random, and 8:2 is the training set to validation set ratio. The network performance is tested using the remaining 25 photos as the test set. The original root dataset used to train the semantic segmentation network is composed of a total of 53,193 images, each measuring 512 × 512 pixels, which are taken from the training and validation sets.

### The restructure root dataset

This paper uses EnlightenGAN to reconstruct the root, randomly selects 5 pictures from the original root dataset, and puts them into EnlightenGAN for 1,000 iterations, and obtains a picture of the reconstructed root and a grayscale image that only labels the reconstructed root. Through the method of image processing, the pixel gray value of the original root label is set to 1, the pixel gray value of the reconstructed root label is set to 2, and the two are added. We can obtain an annotated image containing the original root and the reconstructed root and set the part of the image whose gray value is equal to 3 to 1. Then, get the annotation of the reconstructed root image, divide the training set and the verification set at a ratio of 8:2, and also divide the training set and the verification set into the size of 512 × 512 pixels through the image processing method, finally obtain the training set and verification set with a number of 2,527. It is regarded as a reconstructed root dataset for transfer learning training of semantic segmentation networks.

### Segmentation network

#### Experimental pipeline

**Fig. 1.**
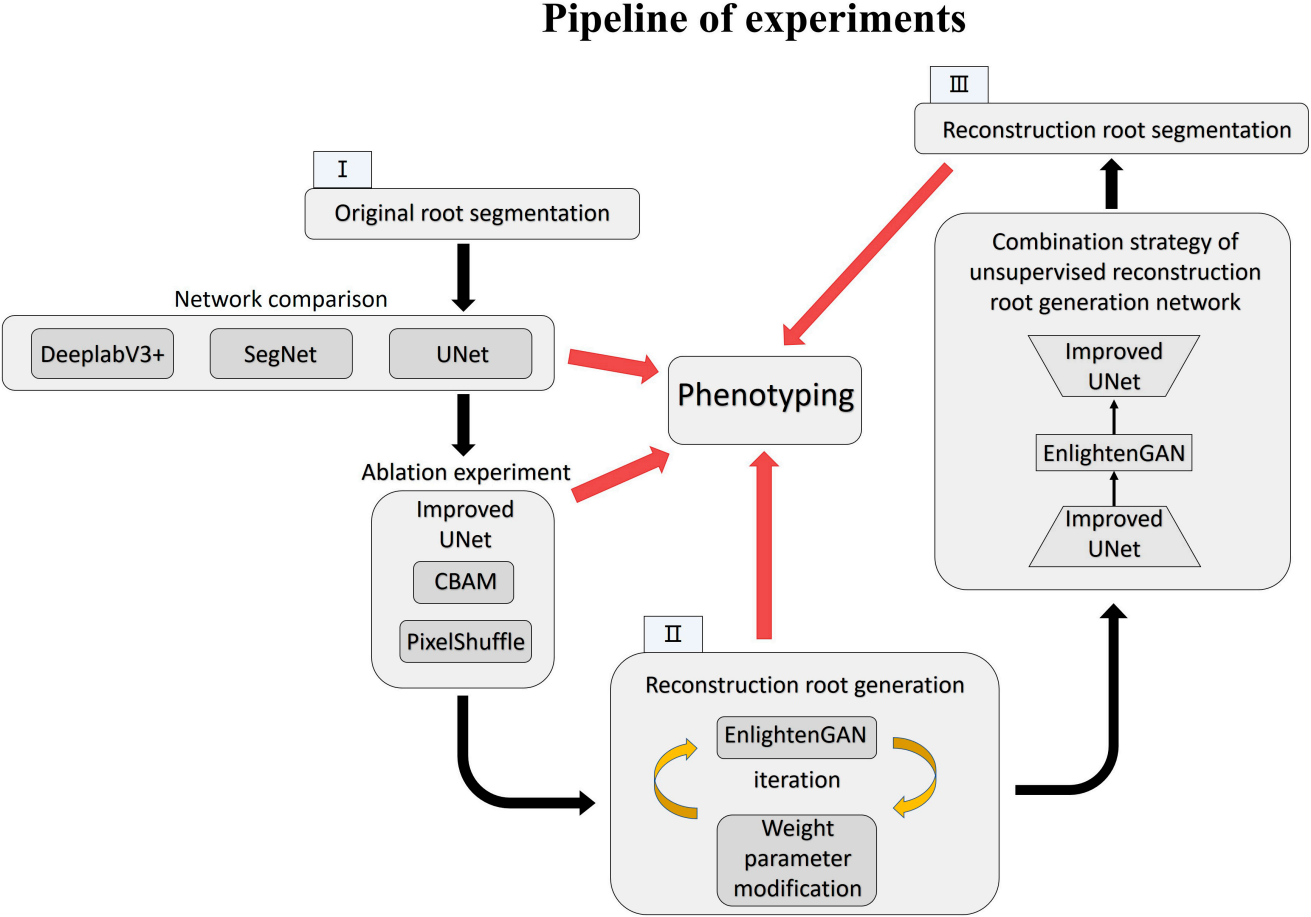
Processes related to the pipeline of experiments.

The general idea of this paper consists of 3 parts (Fig. 1): the first part is the segmentation of the original root. This part mainly examines the segmentation effect of the common semantic segmentation network on the root, improves the network based on their shortcomings, and conducts ablation experiments for comparison. The second part is to reconstruct the generation of the root. This part mainly performs supervised training by modifying the weight parameters of EnlightenGAN. Finally, a more reasonable reconstructed root image is generated. The last part is the segmentation of the reconstructed image, which mainly uses transfer learning to segment the reconstructed root to realize the complete reconstruction and segmentation of the in situ root image.

This article uses the RTX3080Ti (12G) + 16G memory Windows platform for experiments and compares the image segmentation results and network performance of the original root dataset on SegNet, DeeplabV3+, and UNet. Also, we compare the common VGG16 and ResNet backbones in SegNet and UNet networks, compare the MobilenetV2 and Xception backbones in the DeeplabV3+ network, train 100 generations respectively, and take the weight with the lowest training loss for comparison. See Model evaluation for the experimental results.

#### Improvement basis

In the previous research of this experimental group [36,37], it was shown that the CBAM attention module and subpixel convolution have a positive effect on root image segmentation. Therefore, on that basis, this paper uses UNet as the basic network and adds the CBAM attention module and subpixel convolution for improvement.

The CBAM [39] attention module belongs to the channel and spatial attention mechanism. First, the image is input to the channel attention mechanism to determine the recognition object, and then the recognition position is determined through the spatial attention mechanism and finally fused with the original feature map, fusing channel features and spatial features to generate a new feature map. Moreover, CBAM is also a lightweight attention mechanism, which can be integrated into various neural networks, with strong portability and high versatility.

Subpixel convolution [42] uses low-resolution images, reorganizes pixels between convolution and multichannels, and finally obtains high-resolution images. Compared with the traditional upsampling mechanism, subpixel convolution reduces invalid information during upsampling, optimizes gradient operations, and improves pixel accuracy.

**Fig. 2.**
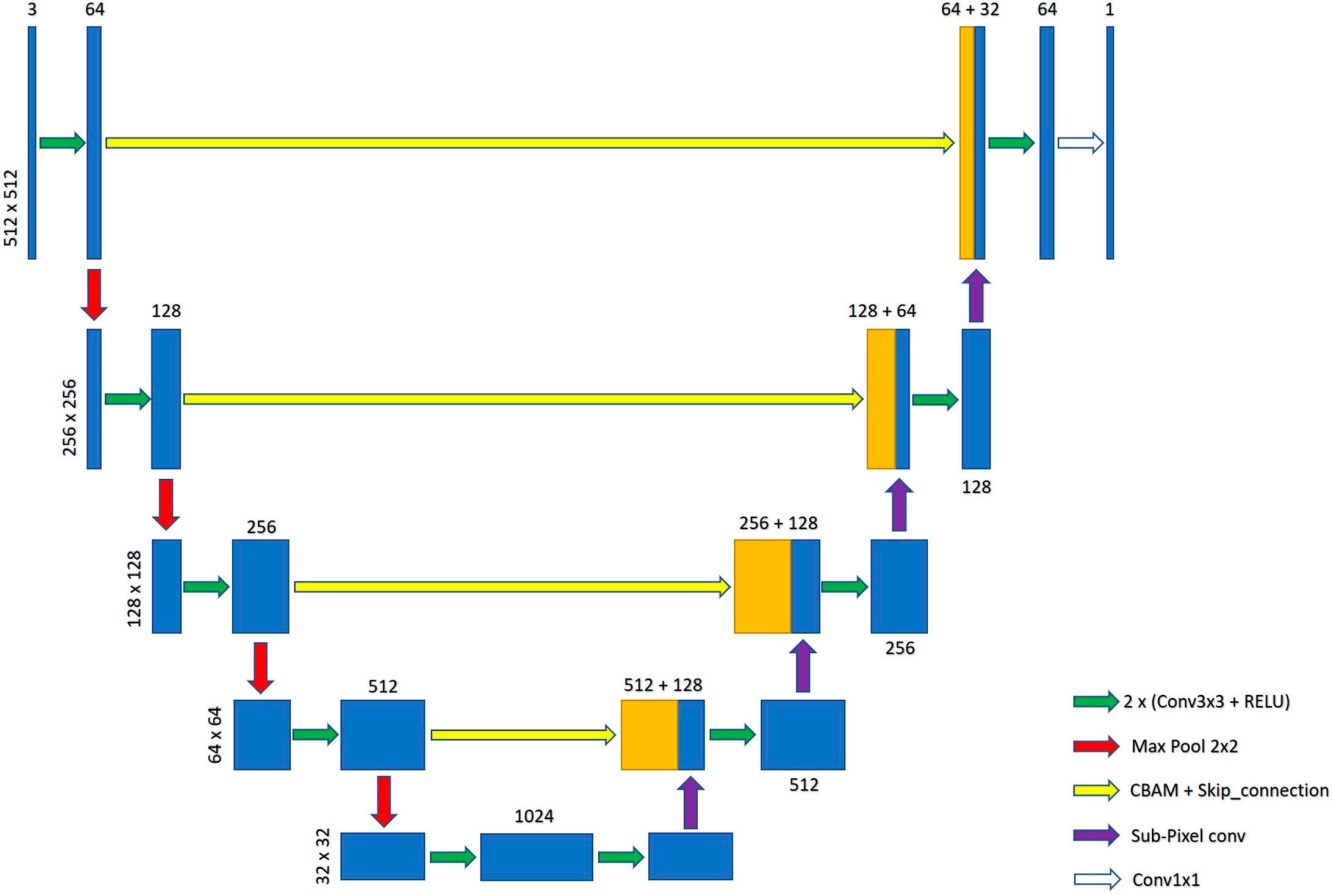
Improved UNet structure.

UNet [27] uses a fully symmetric encoder–decoder structure with skip connections between them for feature fusion. In this paper, there are 2 main points for improving the UNet network: one is to add a CBAM attention module to each layer of skip connections, and the other is to replace the upsampling in the decoder from the original bilinear interpolation to subpixel convolution. In this paper, the PixelShuffle algorithm [42] is implemented, and the corresponding number of channels is changed in the feature fusion part to keep the number of final output channels unchanged. The improved UNet network structure is shown in Fig. 2.

At the same time, we conducted an ablation experiment on the improved UNet network, and the experimental results are shown in the Ablation experiment.

### Restructure network

**Fig. 3.**
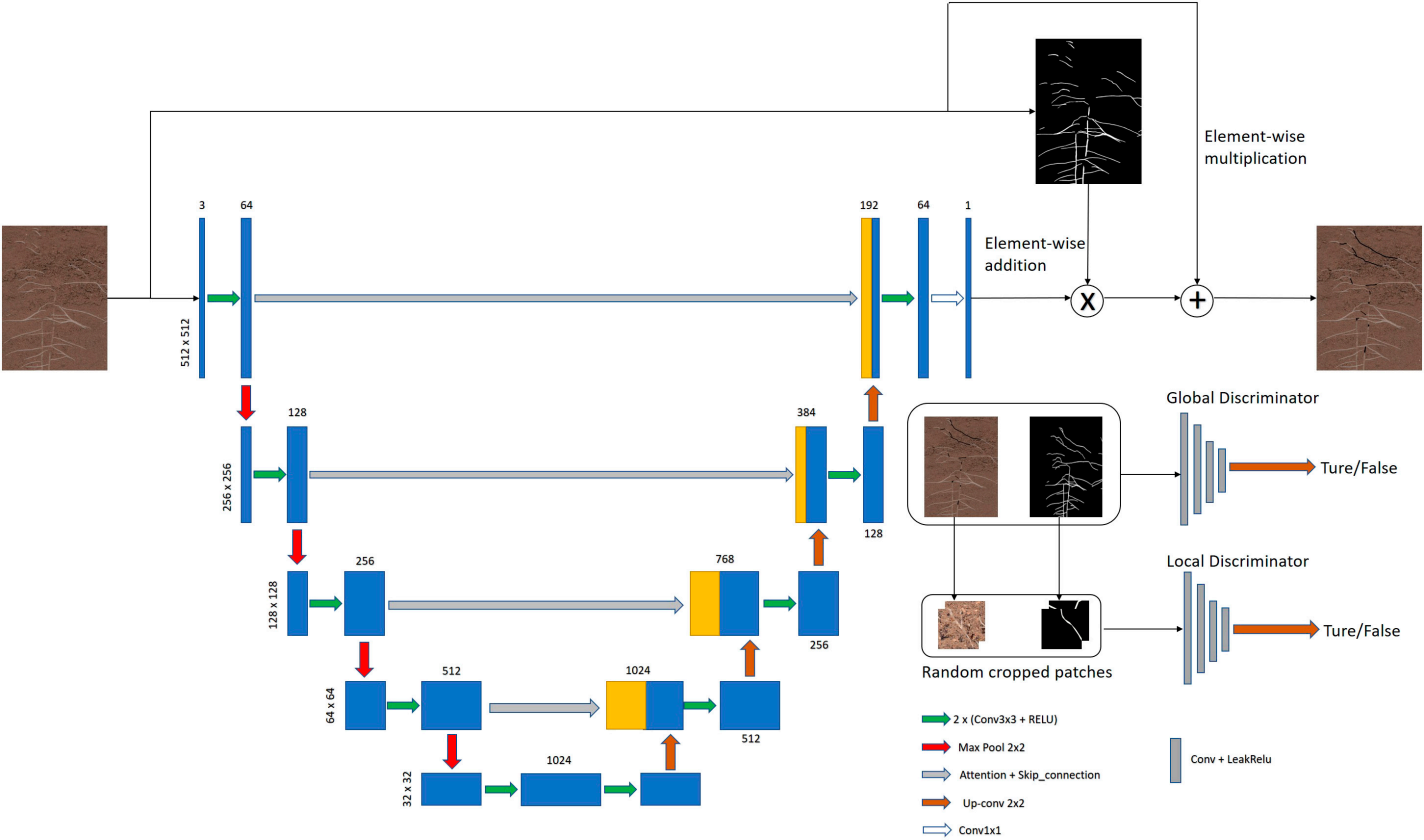
EnlightenGAN structure.

We use EnlightenGAN to construct a reconstruction network. Unlike the unsupervised method of image enhancement methods, root reconstruction uses a supervised method, including 2 channels of trainA and trainB, which input the original root image and labeled image respectively. The principle of EnlightenGAN is shown in Fig. 3. The original root image and annotation are generated by the generator to reconstruct the image of the root system, and the discriminator distinguishes the image from the generator and the segmentation result, respectively. Due to the difference in the gray value of the root system and the background mask, after iterative training, the difference value will be enlarged. At the same time, the image resolution (10,200 × 14,039 pixels) put into the network is much larger than the network requirement (284 × 284 pixels), so when the network is trained on the 284 × 284 pixels mask for brightness adjustment, and resized to match the original image, roots with similar brightness will stick together until the end of the training, and a phenomenon similar to root reconstruction will appear. The number of iterations is positively correlated with the quality of the generated root image. After 1,000 iterations, a relatively complete reconstructed root system can be generated.

### Building the fusion model for roots segmentation and reconstruction

**Fig. 4.**
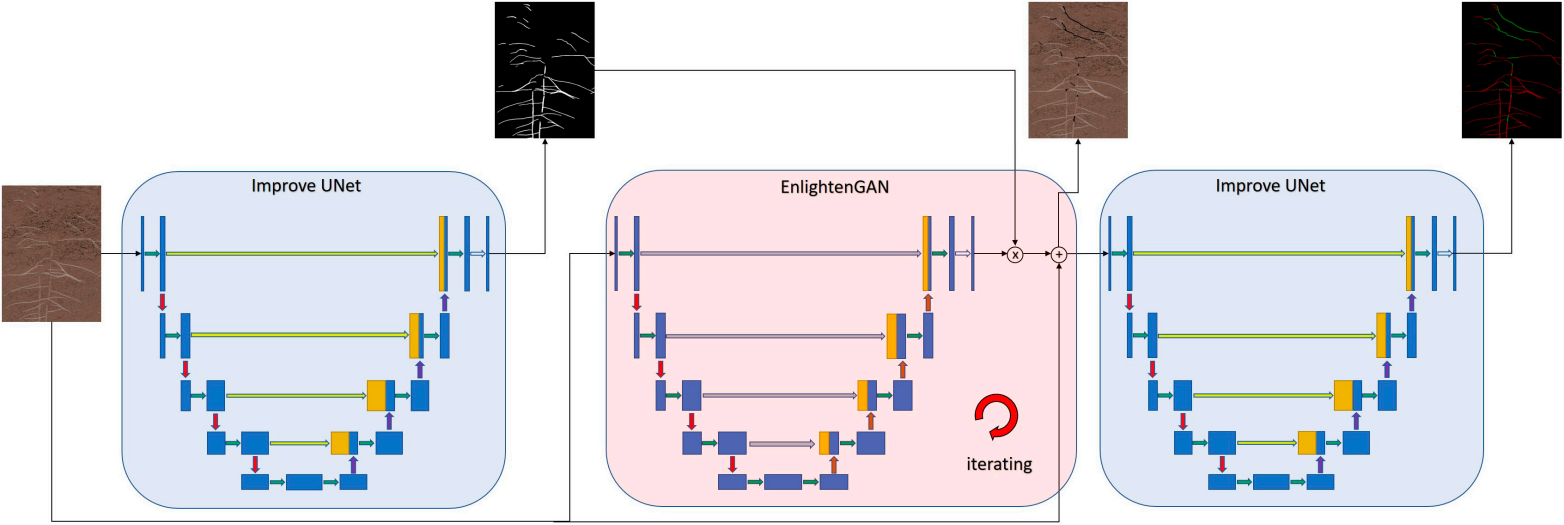
UNet-EnlightenGAN-UNet structure (Discriminator is not listed here).

We combine UNet and EnlightenGAN into a common sequence, as shown in Fig. 4, and that is the structure of UNet-EnlightenGAN-UNet. Results between structures can be exported for different purposes. For instance, only the result of the first UNet is used for general root segmentation; only the result of EnlightenGAN is used for root reconstruction with labels; the result of UNet-EnlightenGAN is used for unlabeled root reconstruction. The EnlightenGAN-UNet result can be used as a marked reconstruction root data expansion method, and the UNet-EnlightenGAN-UNet result can be used as an unlabeled reconstruction root data expansion method.

At the same time, the high-precision improved UNet segmentation results can be used as the input of EnlightenGAN to realize the unsupervised self-learning of EnlightenGAN root reconstruction.

### Evaluation index

#### Segmentation network evaluation index

To objectively and accurately evaluate the performance of various networks, this paper uses various performance indicators such as Mean Intersection over Union (mIOU), Accuracy, and F1-score, and their calculation formulas are as follows:

$$\text{mIOU}=\frac{1}{k+1}\sum_{i=0}^{k}\frac{P_{\mathrm{ii}}}{\sum_{j=0}^{k}P_{\mathrm{ij}}+\sum_{j=0}^{k}P_{\mathrm{ii}}}$$ (1)

$$\text{Recall}=\frac{\mathrm{TP}}{\mathrm{TP}+\mathrm{FN}}$$ (2)

$$\text{Precision}=\frac{\mathrm{TP}}{\mathrm{TP}+\mathrm{FP}}$$ (3)

$$\text{Accuracy}=\frac{\mathrm{TP}+\mathrm{TN}}{\mathrm{TP}+\mathrm{FN}+\mathrm{FP}+\mathrm{TN}}$$ (4)

$$F1=2\times \text{Precision}\times \frac{\text{Recall}}{\text{Precision}+\text{Recall}}$$ (5)

In Eq. 1, the subscript i represents the real value, and j represents the predicted value. In Eqs. 2 to 4, TP is the number of pixels predicted to be the root and actually the root, *FN* is the number of pixels predicted to be the background but actually the root, *FP* is the number of pixels predicted to be the root but the background, and *TN* is the number of pixels that are predicted to be the background and actually are the background. In this paper, these 5 indicators are comprehensively analyzed, and the model is evaluated using the test sets that have not been used for training.

#### Restructure network evaluation index

**Fig. 5.**
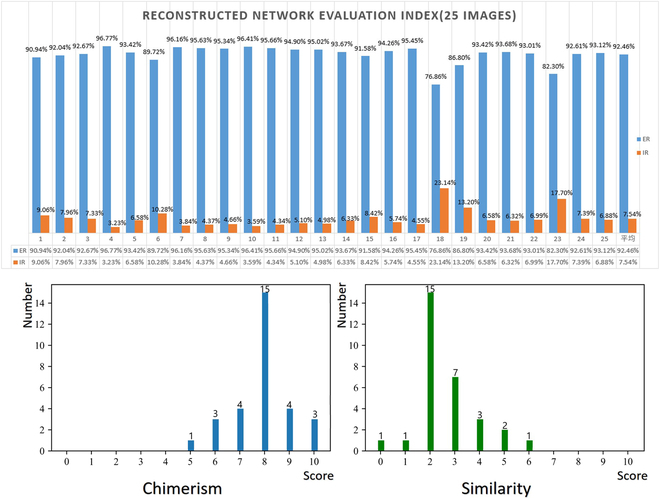
Test set reconstruction root evaluation index (above), and subjective evaluation (below). ER, the proportion of effective reconstruction; IR, the proportion of ineffective reconstruction.

For the EnlightenGAN reconstructed network, there is no standard evaluation index for the reconstructed root. Therefore, we propose an evaluation index based on pixel value, and its operation principles are as follows: 
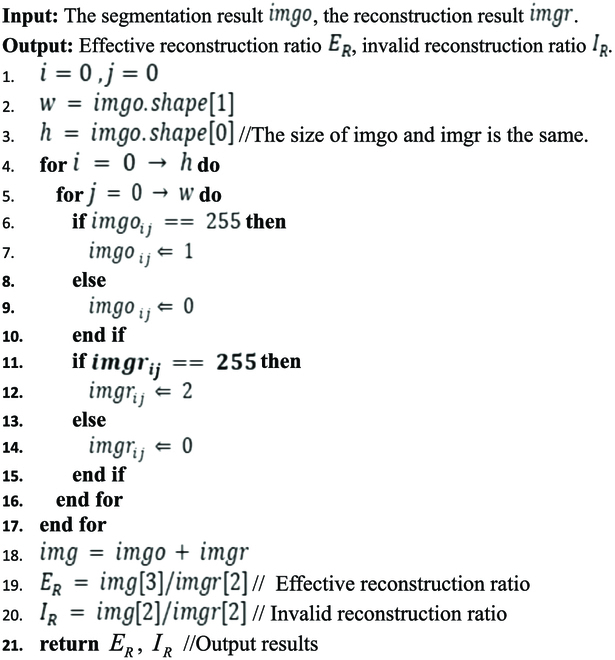


*E_R_* represents the proportion of effective reconstruction, and the closer its value is to 1, the more effective the reconstruction is; *I_R_* represents the proportion of ineffective reconstruction, and the closer its value is to 0, the more effective the reconstruction is. Through statistics and calculations, the effective reconstruction ratio and invalid reconstruction ratio of the reconstructed network can be obtained (Fig. 5, above).

It can be found that in the reconstructed root, the proportion of effective reconstruction can reach 92.46%, and the proportion of ineffective reconstruction is only 7.54%.

At the same time, we interviewed 30 students majoring in agronomy and asked them to rate the reconstructed root. The score components are the chimerism of root reconstruction and the similarity of root reconstruction respectively. The pictures of the test sets were counted (Fig. 5, below). It can be found that most of the students scored very high on the chimerism of root reconstruction. However, the similarity for root reconstruction is not so ideal.

#### Root phenotype evaluation index

In this paper, the 3 indicators of root length, root width, and surface area of each network were measured in the RhizoVision Explorer software [43]. By comparing the Spearman correlation coefficients corresponding to the 3 indicators, the segmentation results of each network for the root were judged. The calculation formula of the Spearman correlation coefficient is as follows:

$$r_{s}=1-\frac{6\sum_{i=1}^{n}d_{i}^{2}}{n\left(n^{2}-1\right)}$$ (6)

By calculation, the Spearman correlation coefficient of the root of each network segment can be obtained. Specific results can be found in Phenotype parameters. It can be found that the improved UNet (ResNet50) network is at the forefront of the comprehensive ranking of phenotype analysis.

## Results

### Model evaluation

#### Model comparison

We compare the segmentation performance of the 3 deep-learning models of UNet, SegNet, and DeeplabV3+ in the original root dataset. The test results prove that the comprehensive performance DeeplabV3+(Xception) > DeeplabV3+(MobileNetV2) > UNet(ResNet50) > UNet(VGG16) > SegNet(ResNet50) > SegNet(VGG16).

**Fig. 6.**
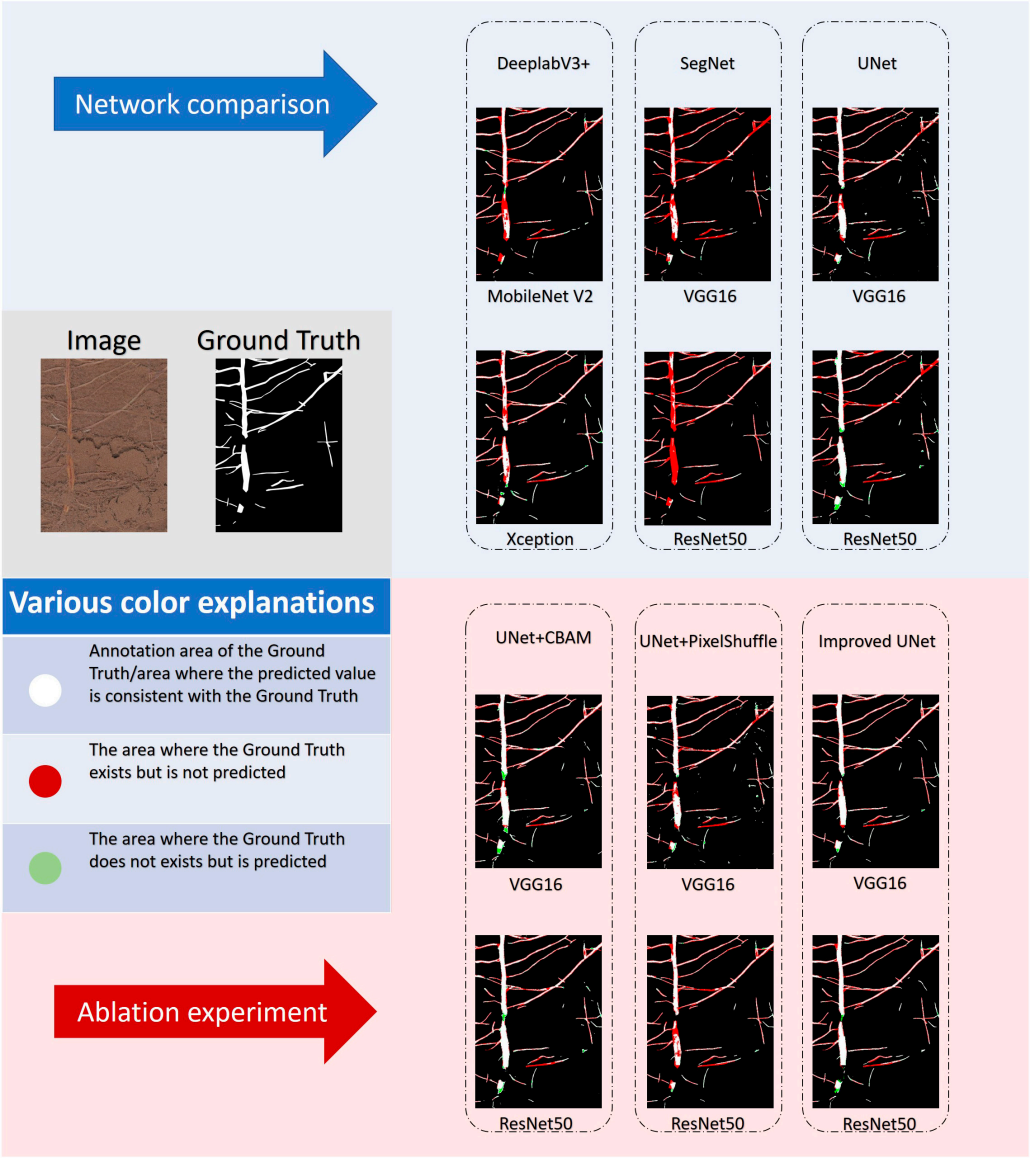
Network comparison (above) and Ablation experiment (below).

As can be seen from the root segmentation image (Fig. 6, above), according to the identification degree of the root, it can be seen that for the root segmented by the SegNet network, the main root is not easy to be identified, and the main root is lost. At the same time, the prediction effect of the lateral root is also not very good. For the root of DeeplabV3+ network segmentation, the prediction effect of the main root is much better than that of SegNet, but some parts are not easy to be predicted. DeeplabV3+ has a good prediction effect on lateral roots. For the root segmented by the UNet network, the identification effect of the main root is very good, but the prediction effect of the lateral root is not ideal. Each of the 3 networks has advantages and disadvantages. Considering the network performance and recognition effect comprehensively, this paper chooses UNet as the base network.

**Fig. 7.**
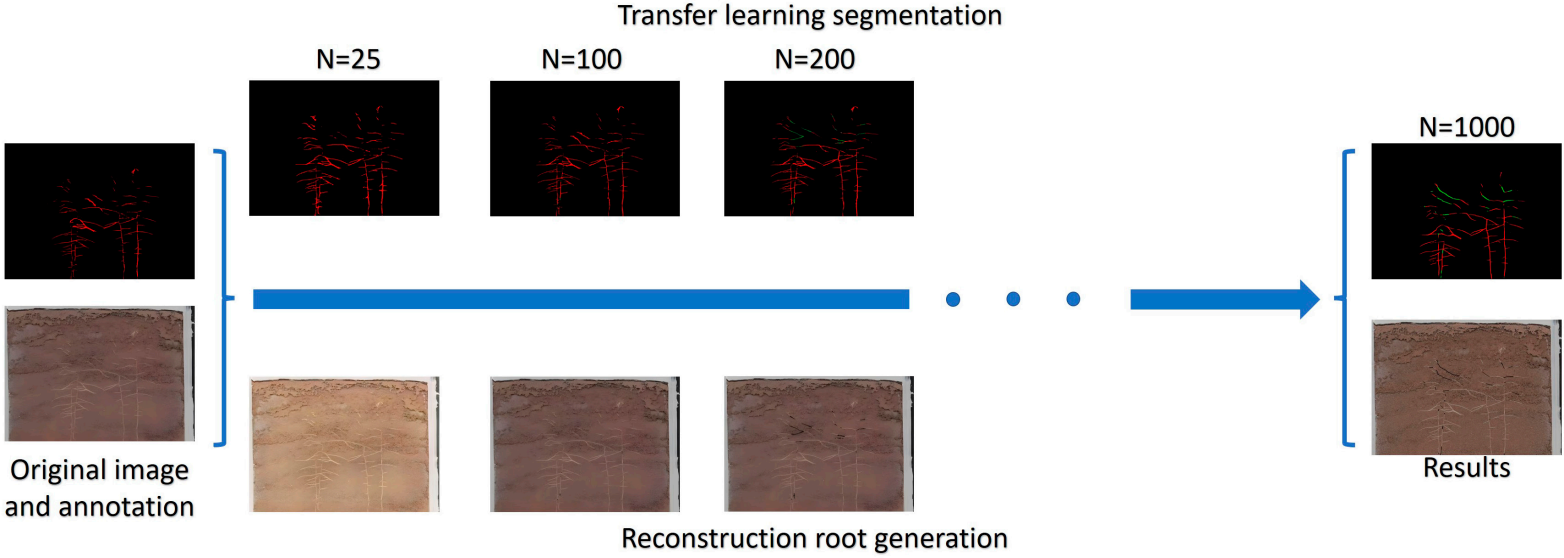
Transfer learning (above) is compared with iterative root generation (below). White (original root), red (reconstructed root).

Through the Improvement basis, we have realized the correction of the disadvantages of the 3 networks, and at the same time, the performance of the network is at the forefront (Table, Network comparison).

**Table.** Experimental result (Network comparison; Ablation experiment; Transfer learning). ρ for and on behalf of Spearman.

Experiment	Network	mIOU	Accuracy(%)	F1(%)	ρ(Length)	ρ(diam)	ρ(surface)
Network comparison	SegNet(VGG16)	83.98	99.03	90.70	0.9617	0.7990	0.8944
	SegNet(ResNet50)	84.07	99.03	90.75	0.9686	0.6874	0.8921
	DeeplabV3+(MobleNetV2)	86.28	99.13	92.13	0.9648	0.7067	0.8959
	DeeplabV3+(Xception)	86.56	99.16	92.31	0.9540	0.8190	0.9221
	UNet(VGG16)	85.33	99.09	91.52	0.8932	0.5343	0.8844
	UNet(ResNet50)	85.33	99.09	92.11	0.9533	0.6551	0.9006
Ablation experiment	UNet+CBAM(VGG16)	86.52	99.17	92.29	0.9386	0.5374	0.9059
	UNet+CBAM(ResNet50)	86.64	99.16	92.36	0.9394	0.6990	0.8982
	UNet+PixelShuffle(VGG16)	85.77	99.12	91.81	0.9648	0.4689	0.8898
	UNet+PixelShuffle(ResNet50)	86.82	99.19	92.49	0.9471	0.6682	0.9283
	Improved UNet(VGG16)	86.73	99.19	92.44	0.9479	0.6767	0.9121
	Improved UNet(ResNet50)	87.03	99.20	92.63	0.9463	0.7567	0.9083
Transfer learning	Improved UNet(VGG16)	82.01	98.66	89.28	0.7977	0.0408	0.9092
	Improved UNet(ResNet50)	82.64	98.71	89.72	0.5238	0.1969	0.7500
Reconstruct root	NAN	NAN	NAN	NAN	0.5800	0.4669	0.7177

#### Ablation experiment

To further verify the recognition effect of various improvement schemes in the improved network on root images, this paper conducts ablation experiments and compares the results of UNet, UNet+CBAM, UNet+PixelShuffle and improved UNet after training on the original root dataset (Table, Ablation experiment).

The results show that when ResNet50 is used as the backbone network, mIOU and F1 are increased by 1.31% and 0.25%, respectively, when the CBAM attention module is added alone, and when subpixel convolution is added alone, mIOU is increased by 1.49%, and F1 is decreased by 0.3%. Try to add the 2 methods to the network at the same time, that is, the improved network in this paper, and find that both mIOU and F1 have a greater improvement, which is 1.7% and 0.52%, respectively. When VGG16 is used as the backbone network, the CBAM attention module is added alone, mIOU and F1 are increased by 1.19% and 0.77%, respectively, and subpixel convolution is added alone, mIOU and F1 are increased by 0.44% and 0.3%, respectively. When the 2 methods are added to the network at the same time, mIOU and F1 are increased by 1.4% and 0.92%, respectively. In the segmentation results of the root (Fig. 6, below), it can be seen that although CBAM and subpixel convolution have a positive effect on network performance, CBAM identifies parts of the soil as roots, and subpixel convolution ignores parts of the roots. When the 2 act at the same time, they can make up for each other's shortcomings. Combining network performance and segmenting images, the improved method used in this paper is more successful.

#### Transfer learning

We use the improved UNet to perform transfer learning on the reconstructed root dataset. By freezing the backbone, changing the number of convolution channels in the last layer, and training for 50 iterations, the results are shown in Fig. 7 (above). The reconstructed root is displayed in red, and the original root is shown in white. The network performance is shown in Table, Transfer learning. A comprehensive comparison of segmented images and network performance shows that the improved UNet has good versatility and robustness.

### Restructure analyze

In this paper, EnlightenGAN is used to realize the generation of the reconstructed roots. The effect and number of iterations of root generation are shown in Fig. 7 (below). When the number of iterations is 25, the root images are enhanced, but no reconstructed roots are generated. There are scattered reconstructed roots beneath the images when the number of iterations reaches 100. After 200 iterations, there are clear reconstruction roots; at 1,000 iterations, a complete reconstruction root is created, and this is when the reconstruction effect is at its greatest.

### Phenotypic parameters

In this paper, RhizoVision Explorer software was used in this study to analyze the results of network comparison and ablation experiments as well as the results of root reconstruction. The total root length, average root diameter, and surface area were calculated for comparison, and the results were obtained through the formula of Spearman correlation coefficient (ρ) to calculate their correlation with the real value, as shown in Table.

In the network comparison and ablation experiments, it was discovered that DeeplabV3+(Xception), SegNet(VGG16), and improved UNet(ResNet50) ranked the top 3 overall, compared with the total root length, average root diameter, and surface area’s Spearman correlation coefficient. In the experiment of root reconstruction and transfer learning, the correlation gap between the reconstructed root and the real value is relatively large, because the reconstructed root connects the broken roots, which will increase the 2 indicators of root length and surface area. At the same time, due to the error of manual labeling and the influence of root hairs, stroke phenomenon will appear in some reconstructed roots, increasing root diameter.

## Discussion

### Model improvement

This study is based on previous studies, the most widely used networks for in situ root image segmentation are SegNet [25], DeeplabV3+ [36–38], and UNet [28,44]. Therefore, this study compares these 3 networks. In the performance comparison, DeeplabV3+ performs the best. When Xception is used as the backbone network, the accuracy reaches 99.16%, mIOU is 86.56%, and F1 is 92.31%; UNet is second. In the case of using ResNet50 as the backbone network, the accuracy is 99.09%, mIOU is 85.33%, and F1 is 92.11%; SegNet again, using ResNet50 as the backbone network, the accuracy is 99.03%, mIOU is 84.07%, and F1 is 90.75%. In the comparison of in situ root image recognition, the 3 have their advantages and disadvantages. Among them, DeeplabV3+ and SegNet have good prediction effects on lateral roots but are not ideal on main roots, while UNet has good prediction effects on main roots but not on lateral roots. Considering the inconsistency of the main root and lateral root morphology, the 3 networks are also inconsistent with the causes of root prediction problems. The reason why the main root is difficult to identify in DeeplabV3+ and SegNet may be that the resolution of the original image is large, and the prediction is to divide the image into several parts for prediction. The details of the main root part are lost. The reason for UNet's poor recognition of lateral roots may be that the network itself needs to be improved in the detailed processing of shallow features, and the network can be improved to enhance the recognition of lateral roots. In summary, this paper chooses UNet as the base network for root segmentation.

After preliminary experimental analysis [36,37], the positive factors of subpixel convolution in root segmentation are clarified. Therefore, based on the UNet network architecture, this paper changes the original upsampling to subpixel convolution and adds the CBAM attention module to the skip connection layer of UNet. Through the comparison of ablation experiments, both modification schemes can improve the performance of the network.

### Stroke phenomenon

Because EnlightenGAN could boost the contrast of the original image, which is easily impacted by elements including ambient light, root hair interference, and labeling errors, the root images reconstruction's stroke phenomena occurred. This phenomenon disrupts the consistency of root architecture and adversely affects root phenotype analysis. Aiming at the reasons for this phenomenon, this experiment proposes several solutions:

1. Given the influence of ambient light, this experiment plans to improve the experimental environment. The RhizoPot used to collect plant root images and the corresponding control terminal are placed in a laboratory with constant temperature and light. The image acquisition is carried out through the automatic acquisition scheme based on the same interval time proposed in the previous research [38] so that the time and space of image acquisition are consistent.
2. Given the influence of root hair interference and labeling errors, the experimental group will employ professional labelers to manually relabel the root and root hairs to minimize the impact of labeling results on reconstruction.
3. EnlightenGan can perform parameter refactoring to reduce root reconstruction errors caused by network parameter problems.

### Reconstructing root and transfer learning phenotyping accuracy drops

Through the experiment of transfer learning, the result of reconstructing root phenotype can be obtained. Since remodeling the root connects the broken roots, the simultaneous stroke phenomenon changes the phenotypic parameters, which reduces the correlation. In transfer learning, due to the emergence of reconstructed roots, the training difficulty increases, so the phenotypic parameters are not as good as the segmentation of the original roots.

### Portfolio strategy

We design a variety of combinations of UNet and EnlightenGAN to achieve different purposes. Its main combinations and uses are as follows:

UNet: Input the original root image and perform accurate segmentation of the root images. Or input the reconstructed root images to perform segmentation of the reconstructed root.

EnlightenGAN: Input the original root images and label them to generate the reconstructed root images, which can be used as an extension of the root dataset (without labels).

UNet-EnlightenGAN: Input the original root images, get the annotation of the original root images, and reconstruct the root images. It can be used as an extension of the original root dataset (with labels) and an extension of the reconstructed root dataset (without labels).

EnlightenGAN-UNet: Input the original root images and labels and get the reconstructed root images and labels. It can be used as an extension of the root dataset (labeled).

UNet-EnlightenGAN-UNet: Input the original root images, get the annotation of the original root images, reconstruct the root images, and reconstruct the annotation of the root images. It can be used as an extension (marked) of the original root dataset and the reconstructed root dataset. It is also possible to change the supervised root reconstruction network to unsupervised.

After modifying EnlightenGAN's parameters to address poor root segmentation results brought on by network issues, the method described in this article can be used as both an expansion of the root dataset and a supplement to the semantic segmentation network. The phenotype analysis of roots has far-reaching significance.

### Generalization

We also use unlabeled wheat images grown by RhizoPot to analyze the generalization performance of the improved UNet network and EnlightenGAN. It proves that the improved UNet network has good generalization performance, and at the same time verifies the feasibility of EnlightenGAN's unsupervised root reconstruction. The segmentation and reconstruction results of the wheat dataset have been uploaded to GitHub (https://github.com/jiwd123/improved_unet)

### Prospect

We compare the root phenotypes of various networks and find that the phenotype parameters of the improved UNet will be closer to the real value than UNet, SegNet, etc., but DeeplabV3+ is the closest to the real value. The reason may be that DeeplabV3+ has added various 2 techniques: dilated convolution, spatial pyramid pooling, and fusion of shallow and deep features. Therefore, the phenotypic parameters of DeeplabV3+ will be closer to the real values. Although the current improved UNet phenotype parameters are not ideal, compared with DeeplabV3+, UNet is more scalable. We intend to keep enhancing the UNet network in the future to bring the segmentation's phenotype parameters closer to the actual value.

The previous results show that when EnlightenGAN reconstructs the root image, the pixel values of the reconstructed root are consistent. This is because EnlightenGAN creates an image of the reconstructed root by altering the target area's contrast, which causes a slight difference between the reconstructed root and the original root. The current workaround involves either using the method of graph neural network to reconstruct the root images of similar areas or replacing the pixel values of the reconstructed root with those of adjacent roots. In this way, the issue of the original root dataset's insufficiency of images can be resolved by using the reconstructed root as an addition to the original root dataset. In the root reconstruction algorithm reported earlier, Thesma et al. [32] used cGAN to generate the root system dataset and then used image processing and other methods to manually reconstruct the root system. Mi et al. [33] used SRGAN to enhance the resolution of the original image and enlarge the details to realize the reconstruction of the root system in the transparent medium. The former one reconstructed root systems by using the image processing method, which is inefficient; the latter one achieved root system reconstruction by enhancing the resolution of SRGAN but cannot reconstruct the root system image of the in situ soil foundation. The advantage of the method proposed in this paper is that EnlightenGAN can automatically generate a reconstructed root system image. Based on meeting the reconstruction accuracy, it improves work efficiency and repairs the broken roots phenomenon caused by soil shading.

Root reconstruction of the main and lateral roots alone has the potential to weaken the stroke phenomenon, and this paper uses traditional image processing methods that can distinguish between main and lateral roots with inconsistent diameters. However, when the main and lateral roots are similar in diameter, they cannot be distinguished. Consequently, we will use the method of deep learning to increase the receptive field of the network to achieve the accurate separation of main and lateral roots in the in-situ root image, thus playing a positive role in root reconstruction.

We proposed an improved UNet segmentation network that combines CBAM and subpixel convolution, designed a reconstruction network based on EnlightenGAN, and discussed the combination strategy of the segmentation network and reconstruction network. The improved UNet achieves 99.2% accuracy, 87.03% mIOU, and 92.63% F1. At the same time, we modified the weight parameters of EnlightenGAN so that it can reconstruct the complete root more accurately. Based on the combination of UNet and EnlightenGAN, this paper discusses 3 segmentation–reconstruction strategies, which can not only generate reconstructed roots for dataset expansion but also convert supervised root reconstruction into unsupervised training. In addition, this paper proposes an evaluation standard for root reconstruction based on subjective visual experience and numerical fusion. According to the subjective perception of the human eye, the reconstructed root's average chimerism score is 7.9, its median score is 8, its average similarity score is 2.9, its median score is 2, and its effective reconstruction rate is 92.46%. It is clear that the experimental approach is excellent for root reconstruction chimerism but less so for similarity. Our future work will focus on improving the generation adversarial network and the segmentation network as well as conducting focused research on the impact of root generation.

## Data Availability

The dataset could be given upon reasonable request from the corresponding author Nan Wang. The code has been uploaded to github: https://github.com/jiwd123/improved_unet.
